# Assessing the Impact of Capture on Wild Animals: The Case Study of Chemical Immobilisation on Alpine Ibex

**DOI:** 10.1371/journal.pone.0130957

**Published:** 2015-06-25

**Authors:** Francesca Brivio, Stefano Grignolio, Nicoletta Sica, Stefano Cerise, Bruno Bassano

**Affiliations:** 1 Department of Science for Nature and Environmental Resources, University of Sassari, Sassari, Italy; 2 Gran Paradiso National Park, Alpine Wildlife Research Centre and Surveillance Service, Torino, Italy; Università della Tuscia, ITALY

## Abstract

The importance of capturing wild animals for research and conservation projects is widely shared. As this activity continues to become more common, the need to assess its negative effects increases so as to ensure ethical standards and the validity of research results. Increasing evidence has revealed that indirect (physiological and behavioural) effects of capture are as important as direct risks (death or injury) and that different capture methodologies can cause heterogeneous effects. We investigated the influence of chemical immobilisation on Alpine ibex (*Capra ibex*): during the days following the capture we collected data on spatial behaviour, activity levels of both males and females, and male hormone levels. Moreover, we recorded the reproductive status of each marked female during the breeding seasons of 15 years. Then, by several a priori models we investigated the effects of the capture taking into account biological factors and changes in environmental conditions. Our results showed that chemical immobilisation did not affect either spatial behaviour (for both males and females) or male hormone levels, though both sexes showed reduced activity levels up to two days after the capture. The capture did not significantly affect the likelihood for a female to give birth in the following summer. Our findings highlighted the scarce impact of chemical immobilisation on ibex biology, as we detected alteration of activity levels only immediately after the capture if compared to the following days (i.e., baseline situation). Hence, the comparison of our findings with previous research showed that our methodology is one of the less invasive procedures to capture large mammals. Nonetheless, in areas characterised by high predator density, we suggest that animals released be carefully monitored for some hours after the capture. Moreover, researchers should avoid considering data collected during the first days after the manipulation in order to avoid biased information.

## Introduction

A wide array of research, conservation and management programmes focusing on wildlife requires the capture and manipulation of animals. Certainly, the development of non- or minimally invasive procedures (e.g., collecting faecal or hair samples for DNA analysis or for the assessment of hormone levels) allows to obtain information without the need to handle animals. Nonetheless, specific information can only be collected by capturing animals (e.g., morphometric measurements, age determination, or blood and serum collection respectively for haematology and for the evaluation of health status of wild population [1,2]). Captures are also important for marking individuals: ecological and behavioural studies, for example, require data collected from individually recognisable animals. New technological advances such as global positioning system (GPS) collars [3], heat sensitive vaginal implant transmitters indicating the birth of neonates [4], and advanced physiological monitoring equipment [5] allow detailed and innovative research on wildlife, but require the capture and manipulation of the animals. Live captures are also required in conservation biology for animal translocations (i.e., reintroductions or population restocking). Moreover, they may represent an alternative to lethal control in management situations in which human safety and properties may be at risk [6].

While the reasons to perform capture of wild animals are clear, researchers have only recently paid attention to assess the risks and negative effects of this activity, particularly in terms of delayed and latent effects. It is a fact that captures may involve risk of mortality, reduction in survival probability [7–9], or injury of focal individuals [10]. Mortality rate is the most important criterion to evaluate the safety level of a capture methodology. In the case of mortality occurring during capture, this rate is rather easy to measure, while delayed mortality (for example, due to myopathy) is much more difficult to estimate [11]. Notwithstanding, mortality cannot stand alone as the measure of capture success and immobilisation safety. The full impact of capture and manipulation procedures cannot be determined without evaluating physical, physiological and behavioural effects on animals at the time of capture and in the days that follow. Indeed, haematological and biochemical blood constituents show that capture and immobilisation of wild ungulates are likely to be some of the most stressful events in their lives [12–14], especially when carried out with snares and nets (e.g., [10]). The duration and magnitude of the stress significantly affect animal physiology and are reflected in an increase in glucocorticoid levels [15]. As glucocorticoids (i.e., cortisol in large mammals) regulate animal behavioural responses [16–19], changes in behavioural pattern related to physiological alterations may also be expected after capture events. Several studies have assessed the effects of capture on behavioural metrics in free ranging wildlife. The potential impacts of different methodologies such as leg snares, clover traps, nets and chemical immobilisation include displacement from the areas surrounding capture sites [20,21], altered space and habitat use [22], depressed movements [10,23,24], and reduction in activity patterns [22].

The effects of immobilisation can considerably differ according to the capture methodology employed. In this regard, relevant published research agrees that captures by remote delivery of immobilising drugs via free-range darting (i.e., chemical immobilisation) lower the animal stress levels, thus decreasing the subsequent capture effects if compared to other techniques (e.g., [10,25–27]). This is likely one of the main reasons why chemical immobilisation is becoming prevalent in large mammal capture [26,28]. Benefits of this approach are: lower mortality and injury rate than other methods [10,27], low capture stress as animals are manipulated while sedated [25,29], and ability to select specific individuals. Conversely, drawbacks include drug-induced side effects depending on the specific drug used (e.g., [30–32]), the fright caused to animals during the approach, and the stress caused by their displacement, which is sometimes necessary in order to perform manipulation procedures (e.g., [23]). By improving capture protocols and adopting improvements in drugs and doses, researchers were able to reduce the mortality rate of this methodology down to minimum levels (e.g., [28,33–36]). However, α2-agonists drugs, such as xylazine and medetomidine, are never completely devoid of toxicity and the induction of sedation invariably carries the risk of severe side effects, e.g., reduction in respiratory rates, hemocytologic and biochemical changes [37], and, in extreme cases, asphyxia due to rumen tympanism [33]. The combination of xylazine and ketamine enables to reduce their dosages, enhances muscle relaxation and duration of effect, and has been associated with faster and smoother induction [38].

Special concern has been raised about the capture and immobilisation of pregnant females, as drugs may reduce reproductive output and survival rate of offspring. Indeed, α2-agonists drugs were observed to affect bovine uterine contractility [39], and repeated doses were reported to cause abortion in horses [40]. Sakamoto et al. [41] found that administration of xylazine to pregnant goats results in an alteration of the physiological parameters of both mother and foetus (i.e., decrease in uterine blood flow caused by the induction of uterine contractions, decrease in circulating blood volume, hypoxemia and acidosis in the mother) that may lead to abortion. In this regard, findings about capture effects on wildlife are not homogenous. Chemical immobilisation decreased productivity of young females in mountain goats (*Oreamnos americanus* [42]), and postnatal calf mortality rates increased after winter immobilisation of pregnant moose cows (*Alces alces*) in Canada [43]. Conversely, in other species no negative effect of chemical immobilisation has been reported on either reproduction or infant survival (e.g., Alpine chamois, *Rupicapra rupicapra* [34]; wild horse, *Equus caballus* [44]; caribou, *Rangifer tarandus* [45]; white-tailed deer, *Odocoileus virginianus* [46]).

Chemical immobilisation can be obtained by approaching animals and shooting a dart from a helicopter, a snowmobile, an off-road vehicle, or simply from the ground. The approach affects the animal stress levels. As a matter of facts, frightened animals have increased heart rate, higher levels of cortisol and other compounds indicating stress and capture myopathy [47]. The approach from the ground likely leads to lower stress levels in wildlife, because animals are generally less frightened. On the other hand, this approach is more difficult to adopt because it requires a closer contact with the animals and high confidence levels with humans. In our study we investigated the influence of capture on the Alpine ibex (*Capra ibex*) population of the Gran Paradiso National Park. Rangers and veterinarians of this protected area experimented the first capture of this species by chemical immobilisation more than 50 years ago [33]. Since then, a hard work has been done in order to increase capture success and to minimise its impacts by improving drug composition and doses, reducing manipulation time and paying maximum of attention to all details which may help cause the least stress possible to animals (e.g., approach only from the ground, manipulation only on sedated animals, no animal displacement). In the light of our literature search, we predicted capture and manipulation to cause an alteration of both ibex movements (aim 1) and activity rate (aim 2) during the hours immediately following capture if compared to the following days. As a consequence of the stress caused to animals by the capture, we expected possible modifications of male hormone levels (aim 3), and in particular of cortisol levels, possibly shrinking over time after the capture event and during the following days. Finally, in order to evaluate possible long-term effects of capture and manipulation procedures on population dynamics, we compared the female productivity of marked females in the years in which they underwent the capture and in the following years (aim 4). To reach our aims, we collected behavioural and physiological information during the time of capture and the following days and we discussed the results by taking into account, case by case, the information on other capture methodologies available in literature.

## Materials and Methods

### Study area

The study took place in the Valsavarenche Valley, within the Gran Paradiso National Park (45°35’N, 7°12’E; North-Western Italian Alps). The study area is a mountainous region with steep glacial valleys ranging from 1500 to 3300 m a.s.l. Rock cliffs, moraines and alpine meadows are the dominant habitats. The vegetation of this area includes conifer woods (*Picea abies*, *Larix decidua* and *Pinus cembra*), scrubs (*Rhododendron* and *Vaccinium* ssp.) and grassland, where the most common grass genera are *Festuca*, *Carex*, *Poa*, *Achillea*, and *Trifolium*. The local climate is temperate, with snowfall mostly occurring from November to April. The warmest period generally occurs from June to September. An automatic station recorded temperature, radiation, precipitation and wind speed data (24 records/d, Property of Meteorological Service of Aosta Valley Region).

Hunting was not allowed in the Park and the presence of a reproductive wolf pack (*Canis lupus*) has been confirmed since 2006. The wolf pack ranged from four to six individuals [48], but no sign of its presence was found in 2014. As regards other relevant natural predators of ibex, the lynx (*Lynx lynx*) has been absent for about a century, while the Golden eagle (*Aquila chrysäetos*) was present in the study area during the whole period of data collection. However, eagles actually had a very limited impact on ibex since they predated only kids of a few months of age. During the 15 years of data collection in this study area, no eagle attack to adult ibex was observed. From 2000 to 2014, the ibex density in the study area ranged from 7.29 to 17.71 ibex/km^2^ (mean ± SD = 12.05 ± 3.48 ibex/km^2^), with a mean sex ratio of 1.03 ± 0.17.

### Chemical immobilisation

Adult ibex were captured between April 2000 and September 2014 as part of an on-going research on the ethology, ecology, and sanitary conditions of the only autochthonous population of this species. The captures were performed by a team with extensive experience in the use of chemical immobilisations with mountain ungulates. The team was composed of at least three rangers and the veterinarian of the Park. To reduce considerable distress in the animals, during the first part of capture only the shooter approached the ibex from the ground with a CO_2_ injection rifle (Dan-inject). The operator measured the distance from the animal by using a laser rangefinder to properly regulate the pressure of the shot of the dart. Animals were darted at a distance of 26.6 ± 6.3 m (mean ± SD), far from cliffs to prevent potentially dangerous situations for the animals and the operators. We chose not to use Hellabrunn mixture for ibex chemical immobilisation [49,50] because of its too high doses of xylazine (125 to 250 mg per animal, up to 3 mg/kg). Instead, chemical immobilisation was achieved with doses of 0.81± 0.08 mg/kg of xylazine and 0.27 ± 0.03 mg/kg of ketamine for male ibex, and doses of 1.95 ± 0.35 mg/kg of xylazine and 0.56 ± 0.10 mg/kg of ketamine for females. Doses were higher for females because their reactivity at the time of the approach and the shooting is always higher than that of males. The endogenous chemical stress mediators act as antagonists of many sedative drugs. Consequently, it was necessary to increase the doses for females to achieve the same sedative effect that was obtained for males. Moreover, females typically use areas closer to rocky slopes than males [51]. Thereby, we needed to induce the sedation of females as rapidly as possible in order to prevent their escape on rocks and to reduce the risk of mortality from falling down.

Once shot, ibex were observed using binoculars. About ten minutes after the injection, the ibex laid down and the capture and manipulation were performed as described in Bassano et al. [33]. We collected biometric data, took biological samples and weighed the animals with a digital scale. Ibex were aged by counting the clearly separated annuli on their horns [52] and ultimately marked. During the manipulation, the ibex were constantly monitored to assess any signs of stress related to capture. Heart rate, respiratory rate, and rectal temperature were recorded. Finally, in order to reverse the effects of xylazine, recover the animals and reduce the risks of hypothermia, we injected 1.0 ml of Atipamezole, a specific alpha-2-adrenergic antagonist; currently, no effective antagonist to ketamine does exist [53]. Ibex were marked with ear tags and visual, VHF (Televilt) or GPS collars (Vectronic Aerospace).

After about 45 minutes, we released the ibex in the same place where they were captured. At least one operator monitored the ibex for about half an hour or at least until the animals reached a safe location and no longer showed any sign of distress. The capture and manipulation protocol was approved by the Italian Ministry of Environment (protoc. n. 25114/04).

### Data collection

From May 2013 to September 2014, we fit 10 males and 9 female ibex with a GPS radio collar (GPS PRO Light collar, Vectronic Aerospace GmbH) set to attempt a relocation every other hour. Moreover, these collars were equipped with an activity sensor that measured activity in two axes based on the actual acceleration experienced by the collar. Activity was measured simultaneously on each axis four times per second as the difference in acceleration between two consecutive measurements, and was given within a relative range between 0 and 255, characterising the mean activity/acceleration [54]. Measurements were averaged over a sampling interval of four minutes and stored with the associated date and time. Localisation and activity data were downloaded by a handheld terminal (VHF connection).

During the capture session in 2013, we collected faecal samples from 9 marked males prior to or during the capture. After the capture, we collected samples twice per day for five consecutive days. We recorded the date and time of collection and stored the samples in a plastic bag at -20°C until the immune assay analysis was performed.

Each summer, we recorded the reproductive status of each marked female captured since spring 2000 (N = 61). The reproductive status was scored with 1 if the female was followed by a kid during June—July and 0 if no kid was present near the female. Variations in female reproductive performances were monitored from the year of capture to the year of their last appearance in our population survey.

### Data analysis

We performed statistical analyses regarding three broad themes: effect of capture on behavioural patterns (movement and activity), male hormone levels and female productivity. To allow for the possibility of a non-linear response to the covariates, we chose to fit Generalised Additive Mixed Models (GAMMs), which are flexible in modelling the shape of non-linear relationships. Moreover, to account for the nested nature of the data, we used ibex identity as a random factor in all models performed. The goodness of fit of each model (homoscedasticity, normality of errors and independence) was checked by visual inspection of residuals. When necessary, the dependent variables were transformed to improve normality of residuals and reduce skew. Statistical analyses were implemented by using R 2.14.1 [55].

### Behavioural patterns

Analysis focused on data (localisations and activity values) collected by the GPS collars during the ten days following the capture. We used localisation data to examine the effect of capture on male and female spatial behaviour. We considered only ascertained localisations and removed any localisation recorded with less than 4 satellites and with a dilution of precision (DOP) greater than 10. With the resulting data, we calculated movement rates (MR, m/h) as straight-line distance (m) between consecutive localisations divided by time interval (h). We transformed MR with a natural logarithm and used it as dependent variable in the models. With data of the activity sensor, we calculated the mean activity values in the same time interval used for MR (2 hours). From these values we calculated the activity rate (AR) as a value on a scale from 0 to 1, dividing it by the maximum value recorded by the collar sensor (255). AR was arcsine square root transformed and used as dependent variable in GAMMs.

The analyses to assess the effects of capture and manipulation on ibex behaviour were performed following the Information-Theoretic Approach [56]. In the light of previous research on related species, theory and biological relevance, we defined a set of alternative hypotheses explaining the variation of ibex movement and activity patterns. Each hypothesis was then translated into a different statistical model that was run and evaluated separately in order to find which model was best supported by the empirical data. As previous studies showed that ibex behaviour is generally influenced by individual and environmental factors [57–60], we included in the models: ibex age and sex, meteorological parameters (i.e., temperature (C°), radiation (W/m^2^), precipitation (mm), wind speed (m/sec)) and, only for MR, altitudinal difference rate between the arrival and the starting location divided by time interval (m/h). Precipitations were calculated as the sum value during the time interval corresponding to each displacement and activity, while radiation and wind speed were calculated as the mean values during the same time interval. Conversely, temperature data were calculated as mean, minimum and maximum values during each time interval. We included sampling time (hour) and day (Julian day) to explain potential daily and seasonal changes in the behavioural patterns. In order to evaluate the effect of the capture event, we used the number of post-capture hours. In the case that the capture affected the behavioural patterns of an individual ibex, we expected a modification (increase or decrease) of MR or AR for a period after capture and, subsequently, the achievement of a plateau, representing the baseline situation (i.e., the ibex behaviour expected with no capture perturbation). We built a correlation matrix (Pearson Correlation Coefficient) within each variable in order to avoid collinearity [61]: only the mean, minimum, and maximum temperature values were collinear. In the analyses we used only maximum temperature because it was better correlated with the response variables (i.e., MR and AR).

For each response variable, we ranked and weighed the alternative models by using the minimum AIC criterion [62]: models with ΔAIC < 2 were considered to be essentially as good as the best model [62]. To avoid retention of overly complex models (i.e., models having additional parameters that result in a minimal increase of fit), we excluded models that simply constituted more complex versions of those with a lower AIC value [63].

### Hormone levels

We extracted steroid metabolites from 0.5 g of well-homogenised wet faecal samples suspended in 5 ml of 80% methanol [64]. Faecal metabolites were measured with validated EIAs, specifically: Epiandrosterone EIA measuring androgens metabolites 17-oxo group for faecal testosterone metabolites [65,66], and 11-oxoetiocholanolone EIA measuring 5 β–androstano-11,17-dione structure for faecal cortisol metabolites [67,68].

We performed statistical analyses of the level of both faecal hormone metabolites (cortisol and testosterone) in two different sets of GAMMs. Only faecal cortisol metabolites were transformed with a natural logarithm in order to improve normality of residuals and reduce skew. As in the analyses on MR and AR, the number of hours after capture was included in the model meant to account for the effect of capture. Moreover, we included the Julian day and male age because these variables may affect the level of hormone metabolites, as shown in a previous study [66].

### Female productivity

Variations in female annual kidding success were analysed by using GAMMs with a Binomial distribution. The response variable was defined as 1 in the years when a female gave birth to a kid and 0 in the other years. The age of the female and the year of data collection were included in the model as continuous variables. In order to account for the effect of the capture event, we also included in the model a variable scored 1 if the female was captured in the current year, 0 if the female was captured in previous years.

## Results

We captured 10 male ibex, aging from 8 to 13 years, between 7 May and 28 June 2013. Nine females (4–15 years old) were captured between 22 April and 16 September 2014. For each marked ibex, during the ten days following their capture we obtained 90.58 ± 8.60 valid relocations, by means of which we calculated MR. The total values of AR of ibex obtained were 112.53 ± 1.47. We collected a total of 83 faecal samples from 9 males (9.11 ± 3.14 for each male), and measured both testosterone and cortisol faecal metabolites levels. From 2000 to 2014, 61 females aging from 2 to 15 years were captured in the study area. During the period of data collection, we recorded a total of 229 observation/year/female in order to analyse their annual kidding success.

### Movement behaviour

**Fig 1:**
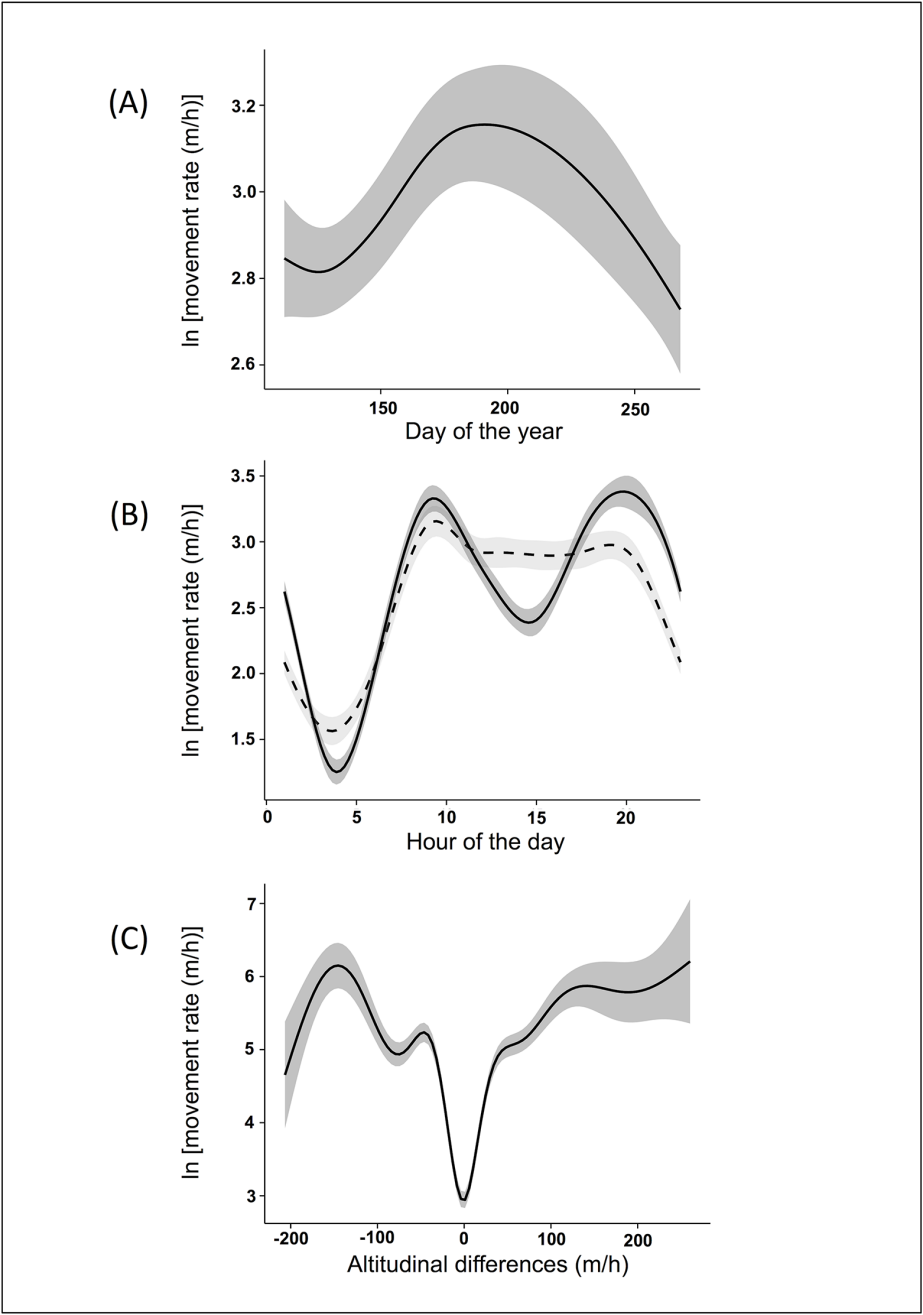
Effects of the dependent variables on ibex movement rate after capture. The values of Alpine ibex movement rate reported were predicted by the best Generalised Additive Mixed Model (see the text for more details) in the Gran Paradiso National Park (Italy) during the ten days following capture. The figure shows the effect of the day of the year (A), the time of day (B) and the altitudinal difference rate (C). In the graph (B), males are represented by continuous line, female by broken line.

The best model (S1 Table) explaining ibex MR included five variables: sex, post-capture hours, interaction between hour of the day and sex, Julian day and altitudinal difference rate. Results of the model showed that ibex MR did not significantly differ between males and females. Moreover, no influence of post-capture hours was detected for both sexes: MR did not change from the time of capture to the following days. The analyses showed a seasonal pattern of variation of ibex MR, which increased from April to the end of June and then decreased until September (Fig 1A). Moreover, the results of the model showed that males and females had different daily movement patterns. Females had the maximum level of MR at 9:30 and slightly reduced their movements later in the day until 20:00. Conversely, males had two distinct positive peaks at about 9:30 and 20:00, and a negative peak during the central part of the day (i.e., 13:00–16:00). Both sexes had minimum levels of MR at about 4:00 (Fig 1B). Finally, the results of the model showed that MR was minimum when ibex moved to areas with little differences in altitude and increased with the increase of the altitudinal difference rate. This increase was particularly evident until reaching an altitudinal difference rate of 50 m/h and then tended to wane with higher values (Fig 1C).

### Activity patterns

**Fig 2:**
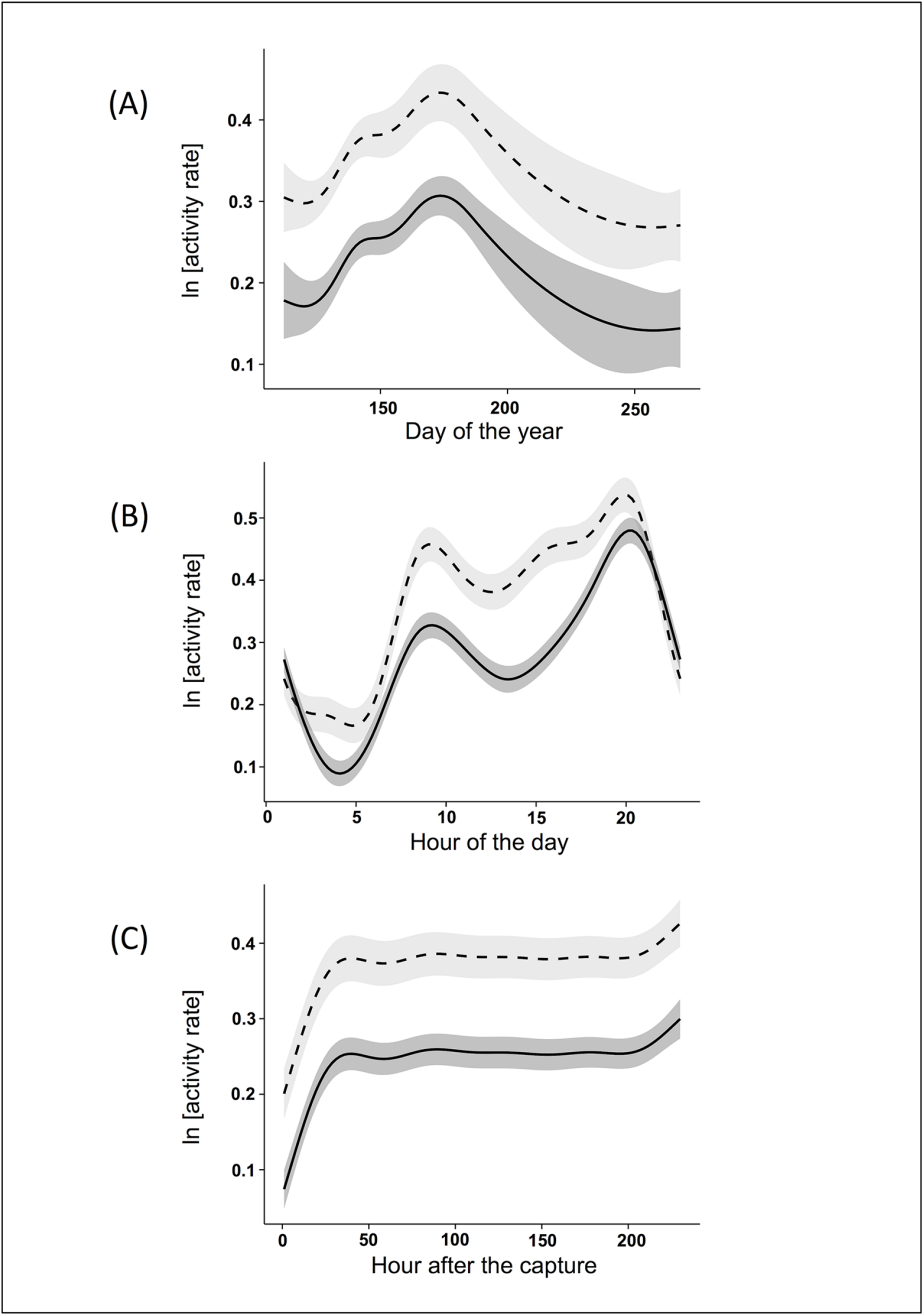
Effects of the dependent variables on ibex activity rate after capture. The values of Alpine ibex activity rate reported were predicted by the best Generalised Additive Mixed Model (see the text for more details) in the Gran Paradiso National Park (Italy) during the ten days following capture. The figure shows the effect of the day of the year (A), the time of day (B) and the hours after capture (C): males are represented by continuous line, female by broken line.

The best model explaining activity patterns (S1 Table) included six predictor variables: sex, post-capture hours, interaction term between sex and hour of the day, Julian day, interaction between maximum temperature and sex, and precipitations. The results of the model showed that females had higher AR than males (β = 0.08 ± 0.02). Both sexes had low AR immediately after the capture. Their AR then increased over time up to about 48 hours, after which the activity appeared to stabilise to a baseline situation (Fig 2C). Seasonal patterns of variation of AR were similar for the two sexes: both males and females increased their AR from April to the end of June and then decreased it until September (Fig 2A). As in the case of MR, the daily pattern of activity was slightly different for the two sexes. Males had two positive peaks of AR at about 9:30 and 20:00 and a negative one at about 4:00. Females showed lower levels of AR during the night (21:00–5:00) and three positive peaks during the day: at about 9:30, 15:00 and 20:00. For both sexes the peak of activity in the evening was higher than that in the morning (Fig 2B). AR linearly decreased with the increase of precipitation levels (β = -0.012 ± 0.003, P<0.001). Both the interactions between age and sex, and between temperatures and sex were included in the best models, though with no significant influence.

### Hormone levels

**Fig 3:**
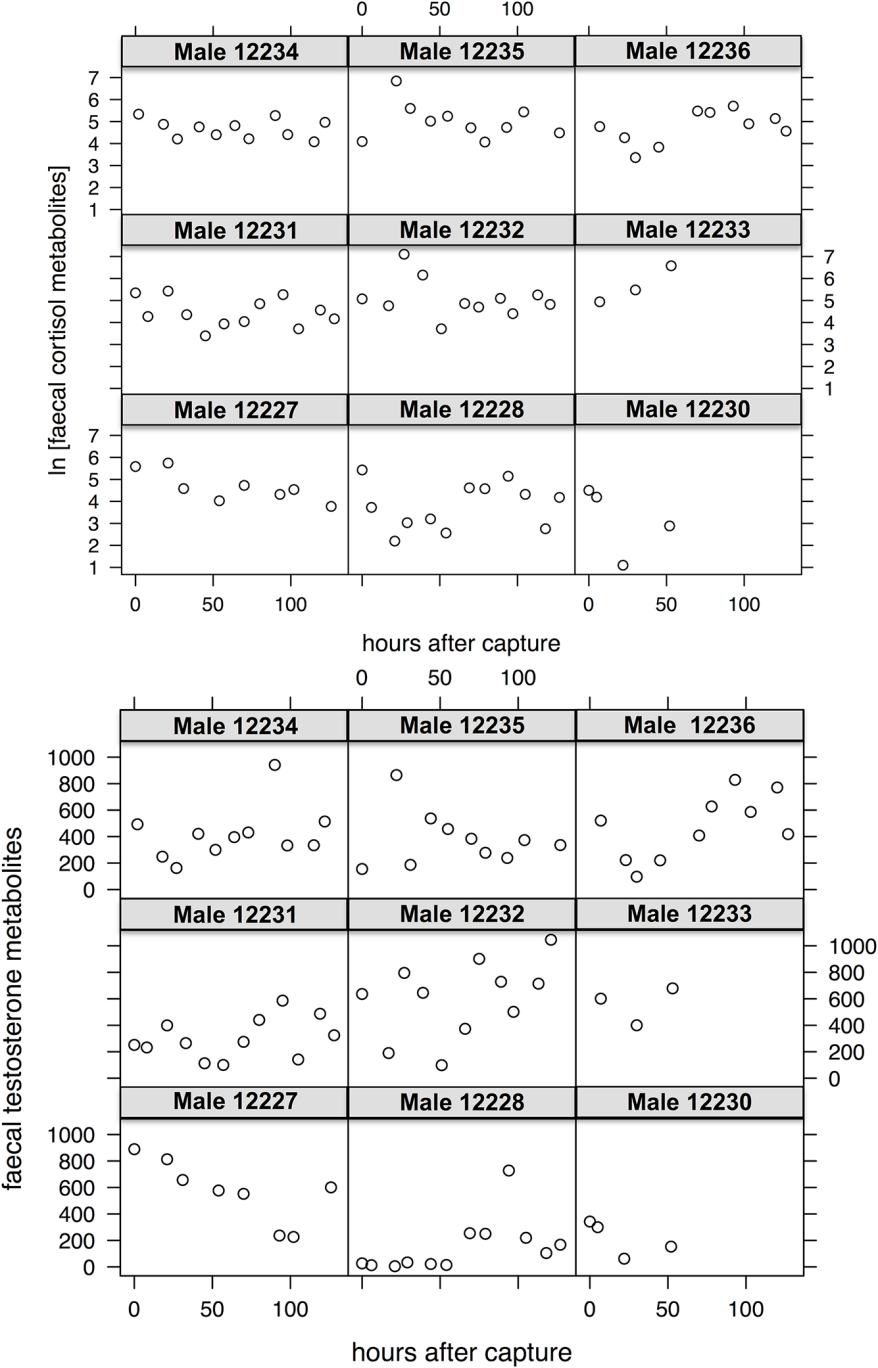
Faecal hormonal metabolites in male ibex after capture. Variation of the values of faecal cortisol metabolites (A) and faecal testosterone metabolites (B) for each sampled male Alpine ibex in the Gran Paradiso National Park (Italy) as a function of the number of hours after the capture event.

Results on hormone levels showed that in male ibex both cortisol and testosterone faecal metabolites did not significantly change with the increase in the number of hours after the capture event, thus indicating no significant effect of capture on their hormone levels. Moreover, the results of the model did not show any influence of the Julian day and male age on both metabolites. Even by looking at individual trends, no significant influence of the capture event on male hormonal excretion was found (Fig 3).

### Female productivity

**Fig 4:**
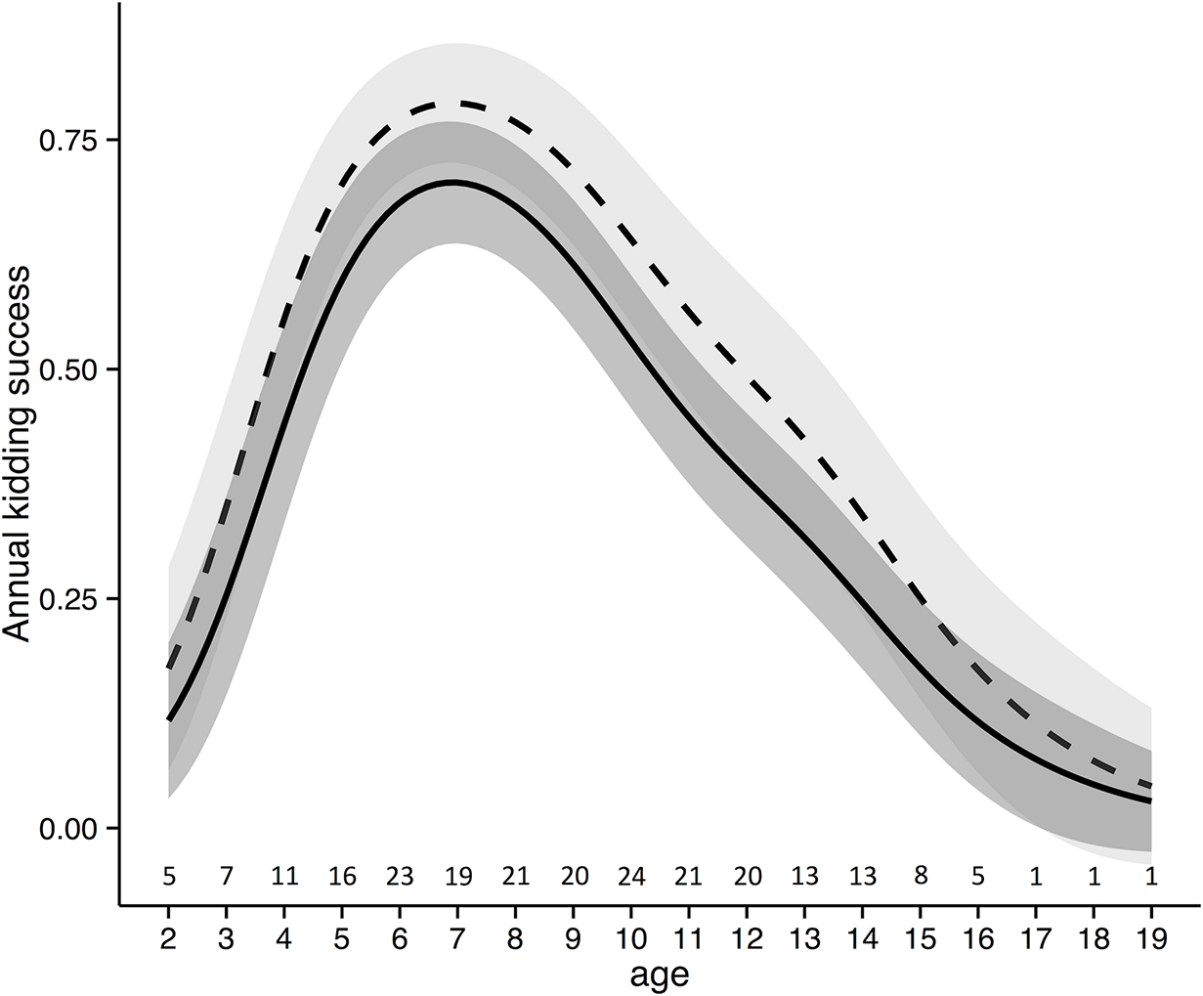
Alpine ibex female productivity in relation to age. The values of female productivity of Alpine ibex reported were predicted by the best Generalised Additive Mixed Model (see the text for more details) in the Gran Paradiso National Park (Italy). The figure shows the effect of age on female productivity in the capture year (broken line) and in the years following the capture event (continuous line).

Results of the model performed to explain female productivity showed that the capture event did not significantly influence the annual kidding success (Fig 4), as the likelihood for a female to have a kid was not significantly different in the birth period following the capture event if compared to the following years. The results of the model showed that the annual kidding success significantly increased from 2000 to 2014 (β = 0.21 ± 0.04). Moreover, female productivity was significantly affected by female age, with a higher kidding success for females of 6–8 years of age and a lower breeding success for younger and older females (Fig 4).

## Discussion

We showed that our capture protocol (i.e., approach from the ground, drug doses reduced and no displacement of animals) had low and short-term impacts on Alpine ibex, with a similar reaction of the two sexes. Indeed, we found alterations with respect to the baseline behavioural patterns only in the short-term analysis of activity levels during the first two days following capture. We did not find any difference in the other short-term effects (spatial behaviour, male hormone levels), as well as in long-term effects (female productivity). Notably, the latter can directly affect life history, animal fitness, and, consequently, population dynamics. In this regard, the analyses of 15 years of female ibex capture activities showed that in our study population the likelihood for a female to give birth, taking into account also age-related effects (see also [69]) and the year-to-year difference in female ibex conditions, was not affected by the capture event (aim 4). These findings are in accordance with DelGiudice et al. [46], Valkenburg et al. [45] and Dematteis et al. [34], who did not find any negative effect of immobilisation by means of xylazine on female reproductive output and offspring survival in white-tailed deer, caribou and Alpine chamois, respectively. Our results, as well as the findings of these three studies, highlighted the importance of drug mixture and doses. As a matter of fact, the combination of xylazine and ketamine, used to immobilise ibex (i.e., our study), white-tailed deer [46], caribou [45] and chamois [34], enables to reduce drug dosages and, consequently, their side effects on the captured animals. Conversely, several studies reported that immobilisation and manipulation may increase foetal loss or early offspring mortality in different large herbivores (e.g., mountain goats [42]; black rhino, *Diceros bicornis* [70]; moose [71]). Such effects, clearly having important consequences on population dynamics, are likely related to drug doses (e.g., 300–330 mg xylazine per animal in mountain goat captures [42]), as well as to the procedures of capture and manipulation (e.g., stress caused by a helicopter chase [70,71]).

In our analyses we took into account some biological and environmental factors that are known to affect ibex behaviour. By including these variables in our models, we managed to account for their effect on ibex behavioural patterns, thus increasing the power of our analyses to detect capture effects and to disentangle them from natural seasonal patterns. In this regard, the results on environmental variables were consistent with previous knowledge on the behaviour of ibex and other ungulate species. We found that MR and AR were highest at the beginning of summer, as it may be expected for herbivores living in an alpine environment where the quality of forage reaches its peak only in this period [72]. Results highlighted peaks of movement and activity during morning and evening hours, as typically found in ungulate species living in temperate regions (e.g., [60,73,74]). Our analyses did not find any influence of meteorological data likely due to the brief duration of the period of data collection.

Analyses of movements showed that, immediately after the capture, both sexes had a MR comparable to that of the following days (aim 1). This finding is partially surprising as we may expect that capture stress urged ibex to adopt a flight behaviour, thus increasing movements away from the capture area. An increase of MR was found in other ungulates species, such as mule deer (*Odocoileus hemionus* [23]) and moose [75]. In these two case studies, the animals were captured by remote darting from helicopter, so the flight behaviour observed in mule deer and moose may be a consequence of the high stress caused to the animals by the helicopter chase. Moreover, Northrup et al. [23] reported that mule deer typically increased MR and daily displacements during the first days after capture in order to go back to their home range, since during capture they were displaced 2–5 km away from their capture site. Conversely, by adopting capture methodologies in which immobilised individuals are not moved, we may expect a reduction of MR, as found in roe deer (*Capreolus capreolus* [22]) and in white-tailed deer [24] which were captured by nets and traps but subsequently not moved away from the capture site. In these two studies, authors found decreased movements after capture lasting for the following 10–14 days. These results were interpreted as the consequence of the need to recover from drug effects (used during manipulation) and rest from the exertion of capture. Conversely, our immobilisation and manipulation procedures did not affect ibex spatial behaviour and therefore appeared to have the least impact on animals, likely as a consequence of the lower stress levels caused by our capture methodology.

The model for activity patterns provided results consistent with our expectations, since the capture affected ibex activity levels (aim 2). The best model assessing AR showed that both sexes were significantly less active after capture. This type of effect was also observed on roe deer by using the same protocol that we used for data collection (i.e., acivity sensor inside the GPS collars [22]). Morellet et al. [22] argued that capture stress accounted for a reduction in the overall activity levels of roe deer in the ten days following the capture event. Ibex needed two days only to regain normal activity rhythms after capture, thus confirming again the reduced impact of our capture methodology if compared to the technique used to capture roe deer. Reduced AR is not in contrast with our findings on spatial behaviour. After capture, ibex moved together with the group of conspecifics. However, in spite of the administration of an antagonist of xylazine, while other ibex fed, the just-captured individuals laid down and slept, thus confirming the long accumulation period of this drug [76]. The just-captured ibex preferred to make an effort and move with other conspecifics, rather than to look for a refuge area and wait until they felt better, as in the case of roe deer [22]. Our conjecture is also supported by several occasional observations made by rangers and members of the capture team. In the hours after the capture, the personnel of the Gran Paradiso National Park usually carries out short and repeated sessions of control of the captured animals by binoculars. During these observations, it is easy to identify the marked ibex within a foraging group because it is often lying on the ground. Our findings highlighted the key role of sociality as antipredator behavioural strategy in response to a stressful event such as captures, manipulation and fitting of a collar. This strategy to move in-group with other conspecifics, though reducing total activity, may be dangerous in areas with a high density of predator population. A lying ibex within a foraging group undergoes higher predation risk in case of attack, for example by a wolf pack. Consequently, the managers should take into account such potential undesirable effect of the chemical immobilisation. In areas characterised by high density of predators, it may be dangerous to release an animal without taking adequate precautions. For this reason, it may be necessary to provide a continuous direct control of the individuals released during the early hours after the capture in order to deter a potential attack by a predator.

The behavioural changes caused by the capture may affect the energy balance of the individuals. As a matter of fact, the reduction in total activity after capture likely results in a reduction in food intake. Blanc and Brelurut [77] reported a decrease of 40% in grazing activity of red deer hinds (*Cervus elaphus*) over a period of eight days after the fitting of a GPS-collar. A similar result was observed directly in red grouse (*Lagopus l*. *scoticus* [78]) and indirectly in bears (*Ursus arctos*, *U*. *americanus*), in which a decrease in body conditions was also detected [10]. Our analyses reported a reduction of total activity, but it is important to consider that ibex gradually returned to a normal activity in about two days. Thereby, it is clear that even though ibex reduced the foraging time for 48 hours, their life history or survival were hardly ever at risk. Moreover, it is useful to note that only during the early hours following the capture the overall activity is very low, while it tends to increase quickly soon after. Hence, the period with a significant reduction in food intake is short and likely to have minor effects on animal fitness.

The analysis about male hormone levels did not show any significant systemic modification (aim 3). As suggested by the increase in faecal cortisol levels (detected in two marked males, Fig 3), the capture and the manipulation caused an acute though short stress. In fact, we were not able to measure any significant change in male hormone levels by using faecal metabolites (cortisol and testosterone). Consequently, we can affirm that chemical immobilisation did not cause serious alterations of hormone levels that may potentially be dangerous for individual wellness. These findings are consistent with the results found for African wild dogs (*Lycaon pictus* [79]) and red colobus monkey (*Procolobus rufomitratus* [80]), in which measurements of faecal cortisol levels indicated no chronic stress in chemically immobilised and marked individuals.

## Conclusions

Our results pointed out that chemical immobilisation, when carefully managed and implemented under strict veterinary control, generated short-term and slightly hazardous behavioural modifications. When possible, we compared our results with those available in literature: overall, the impact of our capture methodology was lower than that of other techniques used to capture large mammal species. As a matter of fact, by comparing our results with those of analogous analyses, we found that alterations of behavioural patterns (i.e., activity and movements) of animals captured with nets or traps persist up to four days or more [22–24,75]. Conversely, ibex showed alterations in activity levels lasting two days only and no significant modification of movements, male hormone levels and female productivity. We argue that such reduced impact of immobilisation is primarily the consequence of several adjustments implemented in order to reduce stress in the captured individuals: (1) we approached the animals and shot a dart at them from the ground, rather than by motor vehicles (e.g., helicopter, snowmobile or off-road vehicle); (2) we used about three times lower doses of xylazine than those described in the literature; (3) we acknowledged the importance of waiting the time necessary after the shooting, as well as of the approach procedures to the sedated animals; (4) we never moved the animals away from the capture site, as in the case of other capture techniques (e.g., [23]). As regards management, our results highlighted the importance of a careful monitoring of captured animals for some hours after their release because of the long-term effects of xylazine, especially in areas with high predator density. For the same reason, we also argue for the implementation of new procedures of sedation, based on the use of more specific α2-agonists drugs (e.g., medetomidine). Finally, we recommend that researchers exclude from their analyses data collected in the first days following capture (two-three days in our case) so as to avoid biased results.

## Supporting Information

S1 Table Top 10 Generalised Additive Mixed Models predicting movement rate and activity rate in Alpine ibex during the 10 days following their capture, in the Gran Paradiso National Park, Italy. X: variables included in each model; logLik: loglikelyhood of each model; AIC: Akaike information criterion; ∆AIC: difference in the AIC value between a given model and the most parsimonious one. See the text for description of predictor variables. In bold the selected models.
(DOCX)
