# Supplementary material for: Assessing the Impact of Capture on Wild Animals: The Case Study of Chemical Immobilisation on Alpine Ibex
Source: PLoS One. 2015 Jun 25;10(6):e0130957. doi: 10.1371/journal.pone.0130957 (PMC4482404; doi:10.1371/journal.pone.0130957)
Supplement: S1 Table — X: variables included in each model; logLik: loglikelyhood of each model; AIC: Akaike information criterion; ∆AIC: difference in the AIC value between a given model and the most parsimonious one. See the text for description of predictor variables. In bold the selected models. (DOCX) [file pone.0130957.s001.docx]

**S1 Table.** Top 10 Generalised Additive Mixed Models predicting movement rate and activity rate in Alpine ibex during the 10 days following their capture, in the Gran Paradiso National Park, Italy.

|  |  |  |  |  |  |  |  |  |  |  |  |  |  |  |  |  |  |  |  |  |  |
| --- | --- | --- | --- | --- | --- | --- | --- | --- | --- | --- | --- | --- | --- | --- | --- | --- | --- | --- | --- | --- | --- |
|  | model # | sex | age | age * sex | hour after capture | hour after capture * sex | hour of the day | hour of the day * sex | Julian day | temperature | temperature * sex | radiation | radiation * sex | precipitations | precipitations * sex | altitudinal difference rate | altitudinal difference rate * sex | **logLik** | **AIC** | **∆AIC** |  |
|  |  | | | |  |  |  |  |  |  |  |  |  |  |  |  |  |  |  |  |  |
| MOVEMENT RATE | 1 | x |  |  | x |  |  | x | x |  |  |  |  | x |  | x |  | -2343.7 | 4715.4 | 0 |  |
|  | **2** | **x** |  |  | **x** |  |  | **x** | **x** |  |  |  |  |  |  | **x** |  | **-2345.8** | **4715.7** | **0.24** |  |
|  | 3 | x |  |  | x |  |  | x |  | x |  |  |  |  |  | x |  | -2346.8 | 4717.6 | 2.19 |  |
|  | 4 | x |  |  | x |  |  | x |  | x |  |  |  | x |  | x |  | -2344.9 | 4717.7 | 2.31 |  |
|  | 5 | x |  |  | x |  |  | x |  |  |  | x |  |  |  | x |  | -2347.0 | 4718.0 | 2.59 |  |
|  | 6 | x |  |  | x |  |  | x | x |  | x |  |  |  |  | x |  | -2343.3 | 4718.7 | 3.24 |  |
|  | 7 | x |  |  | x |  |  | x | x |  | x |  |  | x |  | x |  | -2341.5 | 4719.1 | 3.63 |  |
|  | 8 | x |  |  | x |  |  | x | x |  |  | x |  |  |  | x |  | -2345.7 | 4719.4 | 3.94 |  |
|  | 9 | x |  |  | x |  |  | x | x |  |  |  |  |  | x | x |  | -2343.8 | 4719.6 | 4.15 |  |
|  | 10 | x |  |  | x |  |  | x |  |  |  |  |  |  |  | x |  | -2349.9 | 4719.8 | 4.35 |  |
|  |  |  |  |  |  |  |  |  |  |  |  |  |  |  |  |  |  |  |  |  |  |
| ACTIVITY RATE | **1** | **x** |  |  | **x** |  | **x** |  | **x** |  | **x** |  |  | **x** |  |  |  | **1482.7** | **-2933.4** | **0.00** |  |
|  | 2 | x |  | x | x |  | x |  | x |  | x |  |  | x |  |  |  | 1486.6 | -2933.2 | 0.24 |  |
|  | 3 | x |  | x | x |  | x |  | x | x |  |  |  | x |  |  |  | 1484.2 | -2932.5 | 0.95 |  |
|  | 4 | x |  | x | x |  | x |  | x |  | x | x |  | x |  |  |  | 1487.7 | -2931.4 | 2.05 |  |
|  | 5 | x |  |  | x |  | x |  | x |  | x | x |  | x |  |  |  | 1483.6 | -2931.2 | 2.18 |  |
|  | 6 | x |  |  | x |  | x |  | x | x |  |  |  | x |  |  |  | 1479.3 | -2930.7 | 2.75 |  |
|  | 7 | x |  |  | x |  | x |  | x |  | x |  | x | x |  |  |  | 1485.3 | -2930.6 | 2.82 |  |
|  | 8 | x |  | x | x |  | x |  | x |  | x |  | x | x |  |  |  | 1489.0 | -2930.1 | 3.35 |  |
|  | 9 | x |  |  | x |  | x |  | x | x |  | x |  | x |  |  |  | 1480.8 | -2929.7 | 3.73 |  |
|  | 10 | x |  |  | x |  | x |  | x |  | x |  |  |  | x |  |  | 1482.7 | -2929.4 | 3.99 |  |
|  |  |  |  |  |  |  |  |  |  |  |  |  |  |  |  |  |  |  |  |  |  |

[X: variables included in each model; logLik: loglikelyhood of each model; AIC: Akaike information criterion; ∆AIC: difference in the AIC value between a given model and the most parsimonious one. See the text for description of predictor variables. In bold the selected models.]
